# Altered metabolic pathways elucidated via untargeted in vivo toxicometabolomics in rat urine and plasma samples collected after controlled application of a human equivalent amphetamine dose

**DOI:** 10.1007/s00204-021-03135-8

**Published:** 2021-08-19

**Authors:** Selina Hemmer, Lea Wagmann, Markus R. Meyer

**Affiliations:** Department of Experimental and Clinical Toxicology, Center for Molecular Signaling (PZMS), Institute of Experimental and Clinical Pharmacology and Toxicology, Saarland University, 66421 Homburg, Germany

**Keywords:** Untargeted metabolomics, Toxicometabolomics, Amphetamine, LC-HRMS/MS

## Abstract

Amphetamine is widely consumed as drug of abuse due to its stimulating and cognitive enhancing effects. Since amphetamine has been on the market for quite a long time and it is one of the most commonly used stimulants worldwide, to date there is still limited information on its effects on the metabolome. In recent years, untargeted toxicometabolomics have been increasingly used to study toxicity-related pathways of such drugs of abuse to find and identify important endogenous and exogenous biomarkers. In this study, the acute effects of amphetamine intake on plasma and urinary metabolome in rats were investigated. For this purpose, samples of male Wistar rats after a single dose of amphetamine (5 mg/kg) were compared to a control group using an untargeted metabolomics approach. Analysis was performed using normal and reversed phase liquid chromatography coupled to high-resolution mass spectrometry using positive and negative ionization mode. Statistical evaluation was performed using Welch’s two-sample *t* test, hierarchical clustering, as well as principal component analysis. The results of this study demonstrate a downregulation of amino acids in plasma samples after amphetamine exposure. Furthermore, four new potential biomarkers *N*-acetylamphetamine, *N*-acetyl-4-hydroxyamphetamine, *N*-acetyl-4-hydroxyamphetamine glucuronide, and amphetamine succinate were identified in urine. The present study complements previous data and shows that several studies are necessary to elucidate altered metabolic pathways associated with acute amphetamine exposure.

**Supplementary Information:**

The online version contains supplementary material available at 10.1007/s00204-021-03135-8.

## Introduction

Once introduced as a treatment against narcolepsy, mild depression, post-encephalitic parkinsonism, and several other disorders (Heal et al. 2013), amphetamine nowadays has a limited therapeutic use but is widely consumed as a drug of abuse (DOA) due to its stimulating properties (Carvalho et al. 2012). In 2018, amphetamine was one of the world’s most commonly used stimulants, along with cocaine and methamphetamine (UNODC 2020). In addition to the desired effects such as feelings of energy, sociability, and confidence, many adverse effects including hypertension, tachycardia, anxiety, paranoia or auditory and visual hallucinations are associated with its use (Bonisch and Bruss 2006; Steinkellner et al. 2011). These effects are based on its pharmacological ability to act as an indirect sympathomimetic and to increase the release of different neurotransmitters such as noradrenaline and dopamine and/or inhibit their respective reuptake transporter in the presynaptic membrane (Carvalho et al. 2012; de la Torre et al. 2004). Although amphetamine is consumed since decades, there is still little knowledge available regarding its effects on the metabolic state of the organism (Steuer et al. 2020). Conventional in vitro toxicological studies, e.g., using human dopaminergic differentiated SH-SY5Y cells revealed a neurotoxic effect, which caused mitochondrial dysfunction at a concentration of 3.5 mM (Carvalho et al. 2012; Feio-Azevedo et al. 2017).

Metabolomics in general is used for discovery of novel biomarkers, investigation of physiologic status, or identification of perturbed biochemical pathways (Nicholson and Lindon 2008) and can provide a snapshot analysis of the whole metabolome in a biological system (Liu and Locasale 2017). Toxicometabolomics, a sub-discipline of metabolomics, is dedicated to elucidate the pattern of small molecules (usually below 1500 Da) within an in vitro or in vivo system related to a certain stimulus such as DOA intake. Under highly controlled study conditions, changes of the metabolome can be observed that may indicate or be the result of a certain drug intake (Wang et al. 2016). Toxicometabolomics can, therefore, be used to study toxicity-related pathways such as the mode of action of xenobiotics or in screening of drug induced cellular or organ toxicity, or to discover new biomarkers (Bouhifd et al. 2013; Ramirez et al. 2013). In recent years, toxicometabolomics have been increasingly used in the field of DOA (Araujo et al. 2021; Manier et al. 2020a, b; Steuer et al. 2020; Zaitsu et al. 2016). Its application may allow to find exogenous biomarkers, which could be new drug metabolites, and on the other hand to identify endogenous biomarkers, which could not only be indications of acute drug ingestion or sample manipulation but also provide information in the mechanism of drug action, consumption behavior, or can be used to assess the severity of intoxications (Steuer et al. 2019; Wang et al. 2016). Steuer et al. (2020) investigated changes of the plasma metabolome after amphetamine intake in a controlled human study of 13 participants and identified an increased energy and steroid metabolism. However, since there is no method that can reveal the complete metabolome and since the plasma metabolome is highly dynamic and influenced by various factors, further studies are needed. In vivo studies in laboratory animals are suitable for this purpose. Under well-standardized and comparable conditions such as controlled diet, sleep cycles and little genetic variability, it is possible to better delineate the metabolome changes caused by amphetamine use. Furthermore, to the best of our knowledge, there are no studies on the urinary metabolome after amphetamine exposure available.

This study should provide the metabolic profiling of rat plasma and urine in response to acute amphetamine exposure, provide additional metabolites/biomarker in urine for detection of amphetamine intake and should complement previous studies. Data should allow to observe changes in the metabolome caused by amphetamine and allow to identify biological pathways affected by its intake, which are necessary to further understand its acute and chronic effects and support further targeted analysis. The analysis should be done by liquid chromatography coupled to high-resolution tandem mass spectrometry (LC–HRMS/MS).

## Materials and methods

### Chemicals and reagents

Racemic d-/l-amphetamine sulfate was purchased from Lipomed (Weil am Rhein, Germany). Acetonitrile, ethanol, and methanol (all LC–MS grade) were obtained from VWR (Darmstadt, Germany), ammonium formate, ammonium acetate, and formic acid, amino acids standards solution, d-Glucose-1,2,3,4,5,6,6-d_7_, palmitic acid-d_31_, and creatinine-d_3_ from Merck (Darmstadt, Germany). l-Tryptophan-d_5_ was obtained from Alsachim (Illkirch-Graffenstaden, France). Water was purified with a Millipore filtration unit (18.2 Ω × cm water resistance).

### Study design

Ten adolescent male Wistar rats (Charles River, Sulzfeld, Germany) were housed in a controlled environment (temperature 22 °C, humidity 57 ± 2%, and 12 h light/dark cycles). Studies have been approved by an ethics committee (33/2019—Landesamt für Verbraucherschutz, Saarbrücken, Germany). A single dose of 5 mg/kg body weight (BW) racemic d-/l-amphetamine was administered as aqueous suspension by gastric intubation to five rats. Five control rats were administrated only with water. During the study, rats were housed in metabolism cages for 24 h, having water ad libitum. Animal general health aspects were assessed at the time points 30 min, 60 min, 120 min, 360 min, and 24 h after amphetamine intake. The animals were then monitored including only some general aspects such as body weight, clean orifices, clear eyes, and sleep behavior. Detailed changes expected after intake of stimulants such as heart rate, radial maze for cognitive function or plus maze to determine activity and anxiety behavior were not and could not be monitored as this was not the focus of the current study.

The selected dose of 5 mg/kg BW d-/l-amphetamine is equivalent to 50 mg in a 60 kg human according to the allometric scaling principles of Nair and Jacob (2016). This would correspond to a human d-amphetamine dose of 25 mg, which is in line with the work by Dolder et al. (2017) and 50 mg of a racemic mixture, which is used as recreational drug (http://psychoaktivesubstanzen.de/amphetamin. Accessed 26-May-2020, 9:30).

### Sample collection

Urine was collected separately from the feces over a period of eight or 24 h after administration, aliquoted, and frozen at − 80 °C until use. Blood samples were collected 1, 2, and 8 h after administration. For blood sampling, animals were anesthetized with diethyl ether and blood was withdrawn from the *Vena caudalis mediana* using a heparin-coated syringe. Blood samples were centrifuged (1503 rcf, 5 min, 24 °C), and plasma was removed and immediately stored at − 80 °C until analysis.

### Sample preparation

According to Manier and Meyer (2020), plasma samples were prepared as follow. A volume of 50 µL plasma was transferred into a reaction tube and precipitated using 200 µL of a mixture of methanol and ethanol (1:1, v/v). The mixture contained 48 µM l-tryptophan-d_5_, 8.6 µM creatinine-d_3_, 34.8 µM palmitic acid-d_31_, and 53.4 µM d-glucose-d_7_ as internal standards. Samples were shaken for 2 min at 2000 rpm and subsequently centrifuged for 30 min at 21,130 rcf and 2 °C. 150 µL of the supernatant was transferred into a new reaction tube and evaporated to dryness using a vacuum centrifuge at 1400 rpm and 24 °C for 20 min. The obtained residues were reconstituted in 50 µL of a mixture of acetonitrile and methanol (70:30, v/v).

In accordance with Barnes et al. (2016), urine samples were centrifugated at 13,523 rcf at 4 °C for 10 min to remove any precipitates. 50 µL of urine were transferred in a reaction tube and 200 µL methanol including 48 µM l-tryptophan-d_5_, 8.6 µM creatinine-d_3_, 34.8 µM palmitic acid-d_31_, and 53.4 µM d-glucose-d_7_ as internal standards were added. Samples were cooled to − 20 °C for 20 min and then centrifugated for 10 min at 13,523 rcf and 4 °C. 150 µL of the supernatant were transferred into a new reaction tube and evaporated to dryness using a vacuum centrifuge at 1400 rpm and 24 °C. The obtained residues were reconstituted in 50 µL of a mixture of acetonitrile and methanol (70:30, v/v).

For each matrix and the corresponding timepoint, one pooled quality control (QC) sample was prepared by transferring 10 µL of each sample into one MS vial. These QC samples were also used for optimization of the peak picking parameters and identification of significant features, as described below (QC group).

### LC-HRMS/MS apparatus

According to Manier et al. (2019b), analyses were performed using a Thermo Fisher Scientific (TF, Dreieich, Germany) Dionex UltiMate 3000 RS pump consisting of a degasser, a quaternary pump, and an UltiMate Autosampler, coupled to a TF Q-Exactive Plus system including a heated electrospray ionization (HESI)-II source. Performance of the columns and the mass spectrometer was tested using a test mixture as described by Maurer et al. (Maurer et al. 2018, 2016). Gradient reversed phase (RP) elution was performed on a TF Accucore Phenyl-Hexyl column (100 mm × 2.1 mm, 2.6 µm) and normal phase (NP) elution using a Macherey–Nagel (Düren, Germany) HILIC Nucleodur column (125 mm × 3 mm, 3 µm). The mobile phase and gradient for the Phenyl-Hexyl column consisted of 2 mM aqueous ammonium formate containing acetonitrile (1%, v/v) and formic acid (0.1%, v/v, pH 3, eluent A), as well as 2 mM ammonium formate solution with acetonitrile:methanol (1:1, v/v) containing water (1%, v/v) and formic acid (0.1%, v/v, eluent B). The flow rate was set from 1 to 10 min to 500 µL/min and from 10 to 13.5 min to 800 µL/min using the following gradient: 0–1 min hold 99% A, 1–10 min to 1% A, 10–11.5 min hold 1% A, 11.5–13.5 min hold 99% A. The gradient elution for normal phase chromatography was performed using aqueous ammonium acetate (200 mM, eluent C) and acetonitrile containing formic acid (0.1%, v/v, eluent D). The flow rate was set to 500 µL/min using the following gradient: 0–1 min hold 2% C, 1–5 min to 20% C, 5–8.5 min to 60% C, 8.5–10 min hold 60% C, 10–12 min hold 2% C. For preparation and cleaning of the injection system, isopropanol:water (90:10, v/v) was used. The following settings were used: wash volume, 100 µL; wash speed, 4000 nL/s; loop wash factor, 2. Column temperature for every analysis was set to 40 °C, maintained by a Dionex UltiMate 3000 RS analytical column heater. Injection volume was set to 1 µL. HESI-II source conditions were as follows: ionization mode, positive or negative; sheath gas, 60 AU; auxiliary gas, 10 AU; sweep gas, 3 AU; spray voltage, 3.5 kV in positive and − 4.0 kV in negative mode; heater temperature, 320 °C; ion transfer capillary temperature, 320 °C; and S-lens RF level, 50.0. Mass spectrometry for untargeted metabolomics was performed according to a previously optimized workflow (Manier et al. 2019a, b). The settings for full scan (FS) data acquisition were as follows: resolution, 140,000 fwhm; microscan, 1; automatic gain control (AGC) target, 5 × 10^5^; maximum injection time, 200 ms; scan range, *m/z* 50–750; spectrum data type; centroid. All study samples were analyzed in randomized order, to avoid potential analyte instability or instrument performance to confound data interpretation. Additionally, one QC injection was performed every five samples to monitor batch effects, as described by Wehrens et al. (Wehrens et al. 2016).

Significant features were subsequently identified using PRM. Settings for PRM data acquisition were as follow: resolution, 35,000 fwhm; microscans, 1; AGC target, 5 × 10^5^; maximum injection time, 200 ms; isolation window, 1.0 m*/z*; collisions energy (CE), 10, 20, 35, or 40 eV; spectrum data type, centroid. The inclusion list contained the monoisotopic masses of all significant features and a time window of their retention time ± 60 s. TF Xcalibur software version 3.0.63 was used for data handling.

### Data processing and statistical analysis

Thermo Fisher LC-HRMS/MS RAW files were converted into mzXML files using ProteoWizard (Adusumilli and Mallick 2017). Optimization of XCMS parameter was done on a previously optimized strategy as mentioned by Manier et al. (2019a). Peak picking and alignment parameters are summarized in Table S1 in the supplementary data. Peak picking was performed using XCMS in an R environment (Smith et al. 2006; Team) and the R package CAMERA (Kuhl et al. 2012) was used for the annotation of adducts, artifacts, and isotopes. Feature abundance with a value of zero were replaced by the lowest measured abundance as a surrogate limit of detection and the whole dataset was subsequently log10 transformed (Wehrens et al. 2016). Normalization was performed for urine samples using the area of endogenous creatinine from those samples analyzed using normal phase column and positive ionization mode and for plasma samples using the internal standard l-tryptophan-d_5_. Significant changes of features between control and amphetamine group were assumed after evaluating their fold change using a threshold of 1.5, as well as after Welch’s two-sample *t* test and a *p* value < 0.025. Principal component analysis (PCA) and hierarchical clustering were used to investigate patterns in the datasets. Names for the features were adopted from XCMS using “M” followed by rounded mass and “T” followed by the retention time in seconds. After visual inspection of the extracted ion chromatograms (EIC) of significant features, the significant features were divided into true and false features based on the peak shape quality of their EIC (Hemmer et al. 2020). The R scripts and the mzXML files can be found at https://github.com/sehem/Amphetamine_Metabolomics.git.

### Identification of significant features

Significant features were identified by recording MS/MS spectra using the PRM method mentioned above. After conversion to mzXML format using ProteoWizard (Adusumilli and Mallick 2017), spectra were imported to NIST MSSEARCH version 2.3. Library search for identification was performed using the following settings: spectrum search type, identity (MS/MS); precursor ion *m/z*, in spectrum; spectrum search options, none; presearch, off; other options, none. MS/MS search was conducted using the following settings: precursor tolerance, ± 5 ppm; product ion tolerance, ± 10 ppm; ignoring peaks around precursor, ± *m/z* 1 (Manier et al. 2020b). Following libraries were used: NIST 2014 (nist_msms and nist_msms2 sublibrary) (Linstrom and Mallard 2001), Wiley METLIN Mass Spectral Database (Guijas et al. 2018), LipidBlast (Kind et al. 2013), MMHW (Maurer et al. 2018), the Human Metabolome Database (Wishart et al. 2007) (HMDB, V4.0). Metabolites of amphetamine were tentatively identified by interpreting their spectra in comparison to that of the parent compound. The in-silico fragmentation tool MetFrag (https://msbi.ipb-halle.de/MetFrag/) was applied to MS/MS data to identify potential substructures. Identified features were classified on the different levels of identification according to the Metabolomics Standards Initiative (MSI) (Sumner et al. 2007): affirmation using MS/MS information and co-elution with authentic standards (level 1), affirmation without chemical reference standards, based on comparison of experimental MS/MS spectra with public/commercial spectral libraries (level 2), annotation of putatively characterized compound classes based on characteristic physicochemical properties of a chemical class of compounds, or by spectral similarity to known compounds of a chemical class (level 3), and unidentified or unclassified metabolites (level 4).

### Metabolic pathway analysis

To identify the endogenous metabolic pathways affected by amphetamine intake, all compounds identified with level 1 were imported to MetaboAnalyst 5.0 (http://www.metaboanalyst.ca) and searched against *Rattus norvegicus* metabolite database, for each matrix and time points. Scatter plot was selected as visualization method and the hypergeometric test with the relative-betweenness centrality algorithm was used. For further biological interpretation biochemical pathways with a significant level of *p* < 0.05 was used.

## Results

Data files in mzXML format and the corresponding R files can be found at https://github.com/sehem/Amphetamine_Metabolomics.git. Results of univariate and multivariate statistic as well as the MS^2^ spectra of amphetamine metabolites are available as supplementary data.

### Animal general health aspects

Amphetamine exposed animals in this study showed no effect on their stereotyped behavior or exploratory activity after administration. Furthermore, no significant body weight loss could be observed in comparison to the control group.

### Untargeted metabolomics: univariate and multivariate statistics

Volcano plots of detected features are shown in Fig. S1–4. An overview of the total number of significant features and their percentage of adducts/artifacts, isotopes, and false-positive results are shown in Table S2. In addition, datasets were analyzed using multivariate methods in form of PCA and hierarchical clustering, to identify the largest changing features and specific signatures. Results of the hierarchical clustering which are displayed in heatmaps are shown in Fig. S5–8. Results of the scores of PCA of all matrices and time points are shown in Fig. S9–12.

### Plasma

Using the four different analytical methods (RP positive, RP negative, NP positive, NP negative), 41 features were found in total to be significant at all three plasma time points after amphetamine administration. Plasma samples which were taken 1 h after administration, revealed 14 significant features after using RP and NP and positive ionization mode, which contained one isotope and two adducts according to CAMERA. However, one of these significant features was manually marked as false-positive, due to its EIC showing a poor peak shape quality. Analyses using RP and NP and negative ionization mode did not reveal any significant changes at that time point. Considering the heat maps, a clear separation between the control group and the amphetamine group is shown by NP (Fig. S5a). The dataset of the plasma samples which were taken 2 h after administration, revealed 13 significant features. These features included nine false-positive features, as well as two isotopes. Again, using RP and negative ionization did not reveal any significant features. Looking at the PCA, the two groups amphetamine and control measured in positive ionization mode separated well (Fig. S9b and S11b). In plasma samples received 8 h after administration, 18 significant features were observed only in positive ionization mode. These features included five false-positive hits and two isotopes. Both heatmaps showed a clear separation of the amphetamine and control group (Fig. S5c and S7c).

### Urine

In urine samples, 88 significant features were found in total using the above mentioned four different analytical methods in the samples collected after 8 and 24 h. Sixty-four significant features were found in the 8-h urine samples. These features included 18 false-positive hits, as well as five isotopes and seven artifacts according to CAMERA. Heatmaps showed a good clustering of all groups (Fig. S5d, S6b, S7d, and S8a). Furthermore, in comparison to plasma, amphetamine samples are clustered very closely together in the PCA scores, whereas the control group appears more distributed (Fig. S9d, S10b, S11d, and S12a). Urine samples which were collected 24 h after administration revealed 32 significant features. These features included two false-positive hits, two isotopes, and three artifacts. The four heatmaps displayed a good clustering of the groups (Fig. S5e, S6c, S7e, and S8b). In comparison to the PCA scores after 8 h, the amphetamine group appears more distributed after 24 h.

### Identification of significant features

The results of the identification of significant features are summarized in Tables 1 and 2. The given level of identification was in accordance with the MSI (Sumner et al. 2007). Isotopes that were putatively identified by CAMERA were not further identified. No MS^2^ spectra could be recorded for several features due to their low abundance.

**Table 1:** Identified compounds in plasma samples that showed significant changes between amphetamine (A) and control (C) group, sorted according to compound classes, *m/z* values are given for the highest prevalent ion species

Compound name	Identification level	Compound class	*m/z*	Chromatography	Adducts	Change	*p* (1 h, A vs. C)	*p* (2 h, A vs. C)	*p* (8 h, A vs. C)
Creatine	1	Amino acid	131.0695	RP	M + H	↑	n.s	n.s	*
l-Tryptophan	1	Amino acid	204.0899	RP	M + H, M + H-NH3, M + K*HCOOH, M + 1	↓	**	n.s	n.s
l-Citrulline	1	Amino acid	175.0957	NP	M + H	↓	*	n.s	n.s
l-Histidine	1	Amino acid	155.0695	RP	M + H	↓	**	n.s	n.s
l-Methionine	1	Amino acid	149.0510	RP, NP	M + H	↓	**	n.s	**
l-Proline	1	Amino acid	115.0633	RP, NP	M + H	↓	*	n.s	n.s
l-Threonine	1	Amino acid	119.0582	RP, NP	M + H	↓	**	n.s	*
l-Tyrosine	1	Amino acid	181.0739	RP, NP	M + H	↓	*	n.s	n.s
Amphetamine	1	Amphetamine	135.1048	RP	M + H	↑	n.s	*	n.s
Amphetamine-M (*N*-acetyl)	2 (NIST msms)	Amphetamine	177.1154	RP	M + H	↑	n.s	**	n.s
Ceramide (d18:1/23:0)	2 (Lipidmaps)	*N*-acylsphingosine	635.6216	NP	M + H, M + 1	↑	n.s	n.s	**
Nicotinamide	2 (NIST ms/ms)	Pyridine carboxylic acids	122.0480	NP	M + H	↓	n.s	n.s	*
Tocopheronic acid	3 (hmdb)	Sesquiterpenoids	294.1467	NP	M + H-H2O	↓	**	n.s	n.s
Erucamide	2 (NIST msms)	Unsaturated fatty amide	337.3345	NP	M + H, M + 1	↑	n.s	*	n.s

Identification levels for each metabolite are given according to MSI (Sumner et al. 2007). The corresponding chromatography method is given for normal phase (NP) and for reversed phase (RP) chromatography. Statistical was performed by Welch *t* test (*p* < 0.025): not significant (n.s.) > 0.025

*0.01–0.025

**0.001–0.01

*** < 0.001

**Table 2:** Identified compounds in urine samples that showed significant changes between amphetamine (A) and control (C) group, sorted according to compound classes, *m/z* values are given for the highest prevalent ion species

Compound name	Identification level	Compound class	*m/z*	Chromatography	Adducts	Change	p (8 h, A vs. C)	p (24 h, A vs. C)
4-Hydroxy-6-methyl-2-pyron	2 (NIST msms)		126.0317	NP	M + H	↑	*	n.s
Imidazole lactate	2 (NIST msms)		156.0535	NP	M + H	↑	*	n.s
Histamine	2 (NIST msms)	Amines	111.0796	NP	M + H	↑	**	n.s
l-Pentahomomethionine	2 (METLIN)	Amino acids	219.1293	NP	M + H	↑	*	n.s
l-Tryptophan	1	Amino acids	204.0899	RP	M + H	↓	*	n.s
*N*-acetyl-l-arginine	2 (NIST msms)	Amino acids	216.1222	NP	M + H	↑	**	n.s
*N*-acetylhistamine	2 (NIST msms)	Amino acids	153.0902	RP	M + H	↑	n.s	*
*N*^*2*^, *N*^*5*^-diacetylornithine	2 (NIST msms)	Amino acids	216.1110	RP	M + H	↑	*	n.s
Spermidine	2 (NIST msms)	Amino acids	145.1579	RP	M + H	↓	*	n.s
γ-Glutamyl-γ-aminobutyraldehyde	2 (NIST msms)	Amino acids	216.1110	NP	M-H	↑	**	n.s
Amphetamine	1	Amphetamine	135.1048	RP, NP	M + H-NH3, M + D-NH3, M + H, M + H, M + D, M + 1, M + 2, M + H-107	↑	**	***
Amphetamine-M (3-OH sulfate)	2 (MMHW)	Amphetamine	231.0565	RP, NP	M + H	↑	**	**
Amphetamine-M (4-hydroxy glucuronide)	3	Amphetamine	327.1318	RP	M + H	↑	**	n.s
Amphetamine-M (4-hydroxy-)	3	Amphetamine	151.0997	RP, NP	M + H, M + H-(107), M + D	↑	***	**
Amphetamine-M (6-oxohexanoic acid-)	3	Amphetamine	263.1521	NP	M + H	↑	***	n.s
Amphetamine-M (*N*-acetyl-4-hydroxy glucuronide)	3	Amphetamine	369.1424	RP, NP	M + H, H–H	↑	**	**
Amphetamine-M (*N*-acetyl-)	3	Amphetamine	177.1154	NP	M + H	↑	n.s	*
Amphetamine-M (*N*-acetyl-4-hydroxy-)	3	Amphetamine	193.1103	NP	M + H	↑	*	n.s
Amphetamine succinate	3	Amphetamine	235.1208	NP	M + H, M + D	↑	***	**
5-Acetylamino-6-amino-3-methyluracil	2 (MetFrag)	*N*-arylamides	198.0753	NP	M + H, M + D	↑	*	n.s
1,3-Dimethyluracil	2 (MetFrag)	Pyrimidines	140.0586	NP	M + H, M + D	↑	*	n.s
Urea	1	Ureas	60.0324	RP	M + Na	↑	*	n.s

Identification levels for each metabolite are given according to MSI (Sumner et al. 2007). The corresponding chromatography method is given for normal phase (NP) and for reversed phase (RP) chromatography. Statistical was performed by Welch *t* test (*p* < 0.025): not significant (n.s.) > 0.025

*0.01–0.025

**0.001–0.01

*** < 0.001

### Plasma

In total, 14 compounds could be identified with a level of 1 or 2 (Table 1). 1 h after administration, most identified compounds were amino acids, which could all be identified with level 1 according to MSI. Additionally, the sesquiterpenoid tocopheronic acid was identified. In comparison to the control group all compounds were downregulated. Amphetamine and its metabolite *N*-acetylamphetamine were identified in samples drawn 2 h after administration. Furthermore, erucamide, an unsaturated fatty amide was upregulated compared to the control group. In plasma samples obtained after the 8 h, the identified compounds were again amino acids and *N*-acylsphingosines such as l-methionine and ceramide. While amounts of most amino acids were decreased compared to control group, all other compounds had increased.

### Urine

Table 2 summarizes the 21 compounds which were identified in urine samples. Compared to urine collected after8 h, only amphetamine and its metabolites could be identified in the 24-h urine samples, except for *N*-acetylhistamine. Most of the identified compounds in 8-h urine samples were either amino acids or amphetamine metabolites. All identified compounds had increased in comparison to the control group except for l-tryptophan and spermidine.

### Metabolic pathway analysis

**Fig. 1:**
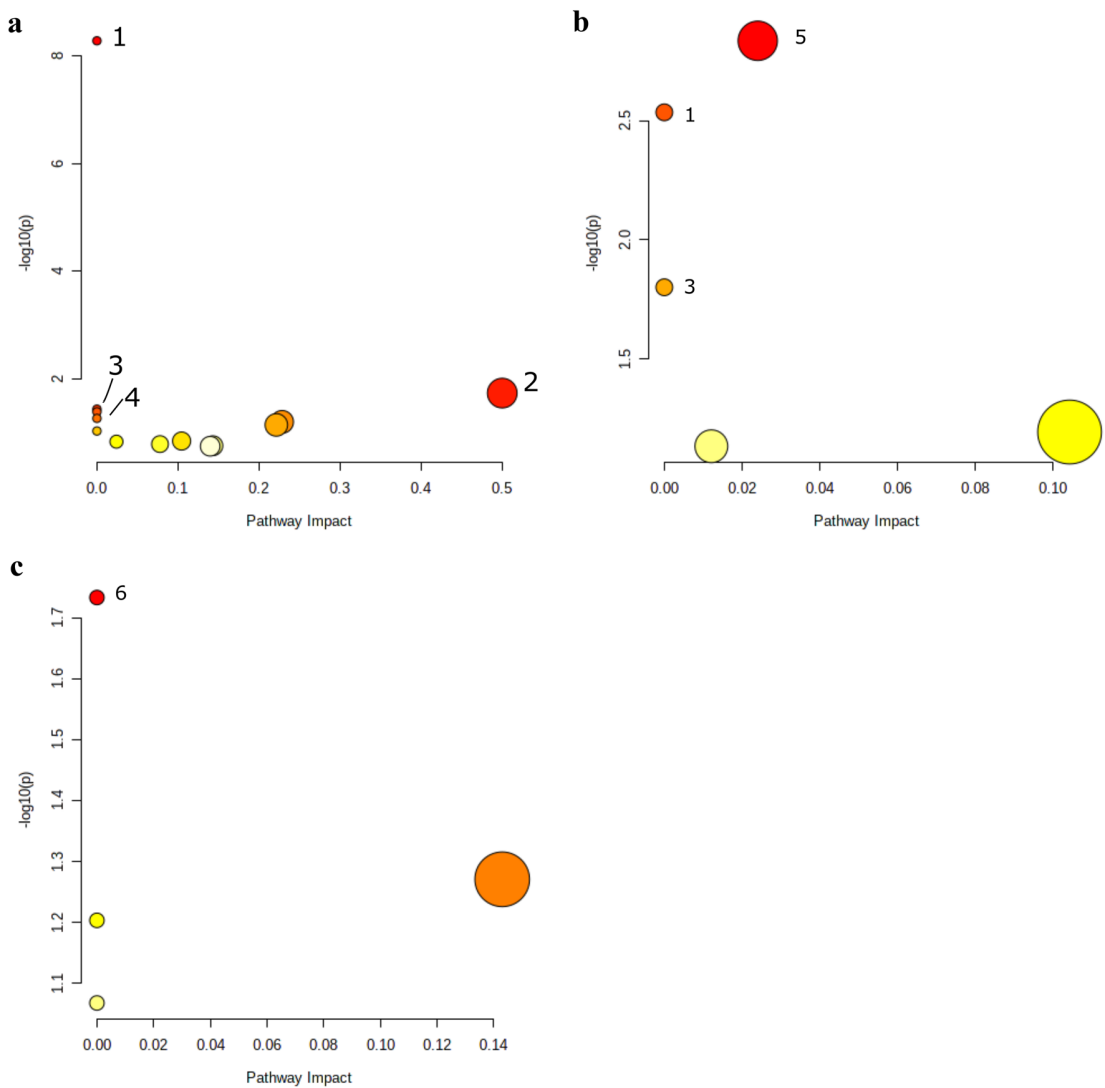
Overview of the scatter plots of the metabolic pathways changed by a single dose of amphetamine (5 mg/kg) in **a** plasma 1 h, **b** plasma 8 h, and **c** urine 8 h after administration. The color of the dots is based on the negative decadic logarithm of the *p* value. Dark color indicates a more significant pathway. The dots radius complies with the pathway impact value. Statistically significant pathways (*p* < 0.05) are numbered from 1 to 6. 1 = aminoacyl-tRNA-biosynthesis; 2 = phenylalanine, tyrosine and tryptophan biosynthesis; 3 = valine, leucine, and isoleucine biosynthesis; 4 = ubiquinone and other terpenoid-quinone biosynthesis; 5 = glycine, serine and threonine metabolism; 6 = arginine biosynthesis

since no substances with a level of 1 were identified in plasma samples 2 h after amphetamine administration, only the scatter plots of 1- and 8-h plasma samples are shown in Fig. 1a, b. The identified metabolic pathway in plasma samples 1 h after administration with *p* < 0.05 were aminoacyl-tRNA biosynthesis, phenylalanine, tyrosine, and tryptophan biosynthesis, valine, leucine, and isoleucine biosynthesis, and ubiquinone and other terpenoid-quinone biosynthesis. For the 8-h plasma samples, glycine, serine, and threonine metabolism, aminoacyl-tRNA biosynthesis, and valine, leucine, and isoleucine biosynthesis were found as significantly changed metabolic pathways. In the 8-h urine samples, only two endogenous metabolites were identified by level 1, therefore, the scatter plot shows only one significant hit for arginine biosynthesis (Fig. 1c). No endogenous metabolites could be identified with level 1 according to MSI in urine 24 h after administration and, therefore, no metabolic pathway analysis was possible.

## Discussion

The metabolome is considered as all compounds with molecular weights less than 1500 Da, which could be detected in, e.g., biofluids or tissues (Barnes et al. 2016). These molecules are not necessarily originating from the biological sample but also from, e.g., tubing vials and reagents. Samples such as plasma or urine are particularly complex since the metabolome is affected additionally by, e.g., food, microbiome, and drugs used to anesthetize experimental animals (Barnes et al. 2016). Since there are many parameters, which can influence the human metabolome, animal models are well suited to study changes in the metabolome, as they are less complex than human studies and can be performed under standardized and comparable conditions. Animals are subject to a uniform sleep–wake rhythm, kept under the same conditions, receive the same food and water, and they have the advantage that their genetic variability is very low. Furthermore, a metabolomic study requires significantly fewer animals than would be needed in a human clinical study to obtain reliable results. They are also beneficial compared to in vitro studies, which often represent only certain cells or organs and thus only a part of an entire organism. Thus, ten male adolescents Wistar rats were used in this study. Certain metabolites are released or excreted into blood and urine due to a certain stimulus such as drug of abuse intake. There they can be identified and serve as potential biomarkers (Wang et al. 2016). While plasma is primarily of interest in terms of changes in endogenous metabolites that may be affected by amphetamine abuse, urine is of interest for detecting metabolites (intake biomarker). The application of untargeted metabolomics to urine may allow the detection of metabolites that may be overseen by conventional pathway analysis methods because they might not be expected. Therefore, both plasma and urine were analyzed in this study to complement and confirm previous studies of the plasma metabolome after amphetamine intake and to detect additional metabolites/biomarker in urine that allow detection of amphetamine abuse. Blood draw time points of 1, 2, and 8 h were chosen to examine both direct and delayed effects of amphetamines on the plasma metabolome. Blood at time point = 0 min was not sampled to avoid additional stress to the animals prior to substance application, which could have influenced the study outcome. Furthermore, individual differences in the animals could be ruled out via the study design as changes in the metabolome between the control and amphetamine group were only assumed to be statistically significant in case they occurred in the complete group. Since the maximum plasma concentration is reached after 15 min, the first two withdrawal time points were 1 and 2 h (Slezak et al. 2018). 8 h after amphetamine administration, amphetamine or metabolites of them could no longer be detected in plasma. However, effects could possibly still be detected but also some changes may also occur at a later time point and thus be undetectable (Gertsman and Barshop 2018). The two urine collection time points were chosen to be able to detect both direct and delayed effects.

It was not possible to find potential reasons for all identified altered metabolites in this study. Conclusions on a pathway can only be drawn if the pathway could be clearly identified by more than one metabolite. Metabolites that occurred as a single phenomenon of a possible pathway must, therefore, first be considered individually in their function. To be able to make specific statements about the influence of the metabolites in association with amphetamine consumption, a targeted study can be considered in which a specific analysis can be made for metabolites that occur in the proposed metabolic pathways.

### Plasma samples collected after controlled amphetamine administration

The complexity of the plasma metabolome was visible comparing the PCA of the plasma datasets to the urine datasets (Fig. S9–12). Urine samples are well clustered in contrast to plasma samples regarding the multivariate statistics. This can be explained by the fact, that in contrast to plasma, most of the identified features in urine belong to amphetamine and its metabolites.

While Steuer et al. (2020) identified in human various metabolites derived from energy metabolism in general, such as acyl carnitines, fatty acids, bile acids, the current study found amino acids to be significantly changed in rat plasma. It needs to be mentioned, that the species may not be directly comparable. The difference in the results between Steuer et al. (2020) and the present study shows that comprehensive studies and different analytical strategies are necessary to study changes within the metabolome. The pathway analysis of time points 1 and 8 h after administration are shown in Fig. 1a and b. Except for creatine, all amino acids were downregulated in the amphetamine-treated rats compared to the control group. The pathway, which was indicated for both time points was the amino-acetyl-tRNA biosynthesis, which is an essential process in protein synthesis (Rubio Gomez and Ibba 2020). While tryptophan, histidine, methionine, threonine, and tyrosine are essential amino acids, proline and tryptophan are functional amino acids, which are important regulators of key metabolic pathways. Such pathways are necessary for maintenance growth, reproduction, and immunity in organism (Wu 2009). In addition to the amino acids, further features were identified, but these belong to MSI level 2 and were, therefore, not included in the pathway analysis. The *N*-acylsphingosine ceramide (d18:1/23:0) was increased in 8-h plasma samples of amphetamine-treated rats. Ceramides are biologically used as membrane stabilizer, energy source and storage, and in inflammatory processes. The observation of amphetamine being able to increase energy metabolism also correlates with other studies conducted both in humans and in rats (Dickson 1998; Tserng and Griffin 2004). Again, species may not be comparable. Another endogenous metabolite, which is associated with the energy metabolism is tocopheronic acid (Fahy et al. 2005; Watson 2006). It is also part of the lipid metabolism and transport and was significantly decreased in comparison to the control group after 1 h of drug administration. Furthermore, nicotinamide was downregulated in amphetamine-treated rats. It is involved in the nicotinamide adenine dinucleotide (NAD^+^) signaling pathway. NAD is synthesized from both nicotinamide and degradation products of the amino acid tryptophan (Canto and Auwerx 2011). It has an important role as a cofactor in numerous metabolic processes such as glycolysis, citric acid cycle of cellular respiration, or other cellular functions (Belenky et al. 2007; Ying 2006). In plasma collected after 2 h, only three features were identified. Two of them were identified as amphetamine and its metabolite *N*-acetyl-amphetamine. The third feature identified was erucamide, which is an endogenous metabolite that causes reduced mobility and slightly decreased awareness in rats (Cravatt et al. 1995; McKinney and Cravatt 2005). Such oleamides could also be originating from disposable laboratory plasticware. To test whether this metabolite was a contaminant from laboratory plasticware or whether it was endogenous in origin, a study was performed according to McDonald et al. (2008) by replacing plasma with methanol. The result showed that erucamide was also found in methanol samples, but compared to plasma, the intensity and peak area was much lower. Additionally, the EIC showed a higher signal in amphetamine-treated plasma than in the control group. Therefore, it might be possible that erucamide was mainly derived from an endogenous source. All identified features except of amphetamine and its metabolite *N*-acetylamphetamine were of endogenous origin and may help to understand acute and long-term effects of amphetamine abuse and are an important complement to already published results.

### Urine samples collected after controlled amphetamine administration

Compared to other biofluids such as plasma, urine is characterized by being easy to collect, rich in metabolites, and able to reflect imbalances in all biochemical pathways within an organism (Khamis et al. 2017). It is, amongst others, also well suited for identifying novel exogenous drug metabolites or endogenous biomarkers indicative for drug ingestion so far, they are not exclusively excreted into feces. This is of particular interest for compounds, which show relatively small detection windows such as amphetamine (Carvalho et al. 2012; Kraemer and Maurer 2002; Musshoff 2000). Therefore, an untargeted metabolomics approach was used in the present study to detect endogenous and (new) exogenous metabolites in rat urine. According to previous studies in mammals (Caldwell 1976; Cho and Wright 1978; Musshoff 2000), expected amphetamine metabolites were formed mainly through (1) hydroxylation in position 4 of the aromatic ring, followed by conjugation of the phenol group with sulfate or glucuronic acid and (2) *N*-deamination and oxidation into corresponding benzoic acid derivatives which were further conjugated with glycine and excreted as hippuric acids (Kraemer and Maurer 2002). However, there might be species differences to be considered. In the present study, seven amphetamine metabolites were found amongst them 4-hydroxyamphetamine and its sulfate and glucuronic acid conjugates. Features, which belong to the *N*-deamination and oxidation pathway were not indicated as statistically significant. However, six metabolites/adducts could be identified (MS^2^ spectra shown in Fig. S13). In detail, the *N*-acetylation, which was also found in plasma samples, the *N*-acetylation together with the hydroxylation of the aromatic ring, the glucuronic acid conjugate of *N*-acetyl-4-hydroxyamphetamine, and the conjugate with acid succinic acid. The 6-oxohexanoic acid adduct cannot be explained from a biological point of view. It is possible that this adduct originated exogenously. However, to our knowledge this is the first report of amphetamine bound to succinic acid in rat. In addition to the amphetamine metabolites mentioned above, endogenous metabolites were also detected in urine. These included metabolites, which belong to the histamine metabolism such as *N*-acetylhistamine and histamine itself. Histamine is a powerful vasodilator, stimulant of gastric secretion, and also a centrally acting neurotransmitter. Furthermore, histamine has a considerable impact on mitigating stress-induced adverse effects in rats (Chen et al. 2020). This observation suggests that amphetamine induces additional stress to rats compared to the control group. Spermidine was decreased in amphetamine-treated rats. Polyamines such as spermidine and spermine play important roles in mammalian cells in protein and nucleic acid synthesis, protection from oxidative damage, activity of ion channels, and cell proliferation, differentiation and apoptosis (Pegg 2016). The pathway which was indicated for urine collected 8 h after administration was the arginine biosynthesis (Fig. 1c). Arginine, a semi-essential amino acid, is synthesized from citrulline, which has also been detected in plasma and is metabolized either to ornithine and urea or to citrulline and nitric oxide (NO). The arginine derivatives *N*-acetyl-l-arginine and urea, and *N*_*2*_*, N*_*5*_-diacetylornithine, a derivate of ornithine, were also detected in urine (Cynober et al. 1995; Sasso et al. 2014). In rats, arginine acts as a key signal for the activation of ureagenesis during high-protein feeding. Additionally, arginine plays an important role in cell division, ammonia-removing from body, immune function, and hormones release. As a precursor of NO, the smallest signaling molecule in mammalian cells, arginine is thus indirectly involved in the regulation of blood pressure (Cynober et al. 1995). γ-Glutamyl-γ-aminobutyraldehyde, imidazole lactate, 5-acetylamino-6-amino-3-methyluracil, and 1,3-dimethyluracil fluctuated significantly in urine. However, the biological significance of these metabolites is currently unclear.

### Limitations of the study

The present study provides only a snapshot of the metabolome in rats and a direct transfer to humans is not possible. Furthermore, individual altered features in this study could only be partly explained in terms of their general function in mammals, but not how they relate to amphetamine abuse. This is due to the fact that it is not possible to draw reliable conclusions about a specific pathway based on a single feature and thus explain processes in the organism. Thus, further studies are needed to draw reliable conclusions. However, the findings of this study may help to first understand the impact of amphetamine on the metabolome of mammals but—and this is much more of relevance—to allow a targeted design of future human studies that need then fewer subjects. Furthermore, the newly detected metabolites in rats may potentially not be formed (at least to this extent) in humans.

## Conclusion

Due to the complexity of the metabolome in plasma and urine with its multitude of different metabolites, it is not possible to establish an untargeted metabolomics approach that allows a holistic view on the metabolome. For this reason, the present study is a further piece in the puzzle to elucidate, which metabolic changes occur in an organism after amphetamine intake. In this study, the major endogenous metabolites that were significantly altered belong to the compound class of amino acids. Furthermore, new amphetamine metabolites *N*-acetylamphetamine, *N*-acetyl-4-hydroxyamphetamine, *N*-acethyl-4-hydroxy-glucuronic amphetamine, and an amphetamine succinic acid conjugate were identified, which may be used for detection of amphetamine intake. The example of the succinate metabolite shows that untargeted metabolomics allows to identify metabolites that would otherwise not have been expected or would not have been searched for in a targeted approach.

## Supplementary Information

Below is the link to the electronic supplementary material.

Supplementary file1 (PDF 4001 KB)

## Data Availability

The R scripts and the mzXML files can be found at https://github.com/sehem/Amphetamine_Metabolomics.git.
