# Supplementary material for: Altered metabolic pathways elucidated via untargeted in vivo toxicometabolomics in rat urine and plasma samples collected after controlled application of a human equivalent amphetamine dose
Source: Arch Toxicol. 2021 Aug 19;95(10):3223–34. doi: 10.1007/s00204-021-03135-8 (PMC8448701; doi:10.1007/s00204-021-03135-8)
Supplement: Supplementary file 1 — Supplementary file1 (PDF 4001 KB) [file 204_2021_3135_MOESM1_ESM.pdf]

Altered metabolic pathways elucidated via untargeted in vivo toxicometabolomics in rat urine and plasma samples collected after controlled application of a human equivalent amphetamine dose

Selina Hemmer[1], Lea Wagmann[1], Markus R. Meyer[1]

[1]Department of Experimental and Clinical Toxicology, Institute of Experimental and Clinical Pharmacology and Toxicology, Center for Molecular Signaling (PZMS), Saarland University, 66421 Homburg, Germany

Electronic Supplementary Material

Table S1. Overview of the peak picking and alignment parameters used for preprocessing for the respective matrices. NP = normal phase chromatography, RP = reversed phase chromatography, pos = positive, neg = negative, ppm = allowed ppm deviation of mass traces for peak picking, snthresh = signal to noise threshold, mzdiff = minimum difference in *m/z* for two peaks to be considered as separate, prefilter 1 = minimum of scan points, prefilter 2 = minimum abundance, bw = bandwidth for grouping of peaks across separate chromatograms.

Matrix, time point	Column	Polarity	Peakwidth, min	Peakwidth, max	ppm	sntresh	mzdiff	Prefilter 1	Prefilter 2	bw
Plasma, 1h	NP	pos	9.9	40	1.0	47	0.038	10	9100	0.5
		neg	7.8	12	1.2	14	0.048	6	3800	0.5
	RP	pos	10.0	10	1.5	100	0.048	15	10000	1.0
		neg	8.9	96	1.0	80	0.002	6	8800	0.5
Plasma, 2h	NP	pos	9.9	22	1.0	26	0.038	9	700	0.5
		neg	9.9	12	1.2	23	0.002	6	6600	0.5
	RP	pos	9.9	11	1.6	96	0.054	16	10000	3.5
		neg	10.0	62	1.0	83	-0.096	1	9000	0.5
Plasma, 8h	NP	pos	10.0	24	1.0	23	0.018	10	9600	0.5
		neg	8.9	10	1.0	74	0.042	8	1900	0.5
	RP	pos	8.9	10	1.6	98	0.010	15	9700	2.1
		neg	9.2	10	1.0	77	0.016	8	9800	0.5
Urine, 8h	NP	pos	9.9	22	1.1	36	0.062	15	300	0.5
		neg	8.9	10	1.2	97	0.038	11	8200	0.5
	RP	pos	10.0	11	1.8	97	0.016	18	500	2.0
		neg	10.0	76	1.2	34	0.044	6	5600	0.5
Urine, 24h	NP	pos	9.9	12	1.0	90	0.090	17	1600	0.5
		neg	8.9	16	1.5	65	0.062	9	7000	0.5
	RP	pos	9.8	12	1.3	61	0.030	19	900	1.5
		neg	9.9	59	1.0	42	0.010	8	300	0.5

Table S2. Overview of the total number of significant features found with all 4 different analytical methods (reversed phase chromatography positive/negative and normal phase chromatography positive/negative) and their percentage of adducts/artifacts, isotopes and false-positive results in the respective matrices. Features found with more than one analytical method count only once.

Matrix	Time point	Number significant features	Adducts/Artifacts	Isotopes	False-positive
Plasma	1h	14	2	1	1
	2h	13	0	2	9
	8h	18	0	2	5
Urine	8h	64	7	5	18
	24h	32	3	2	2

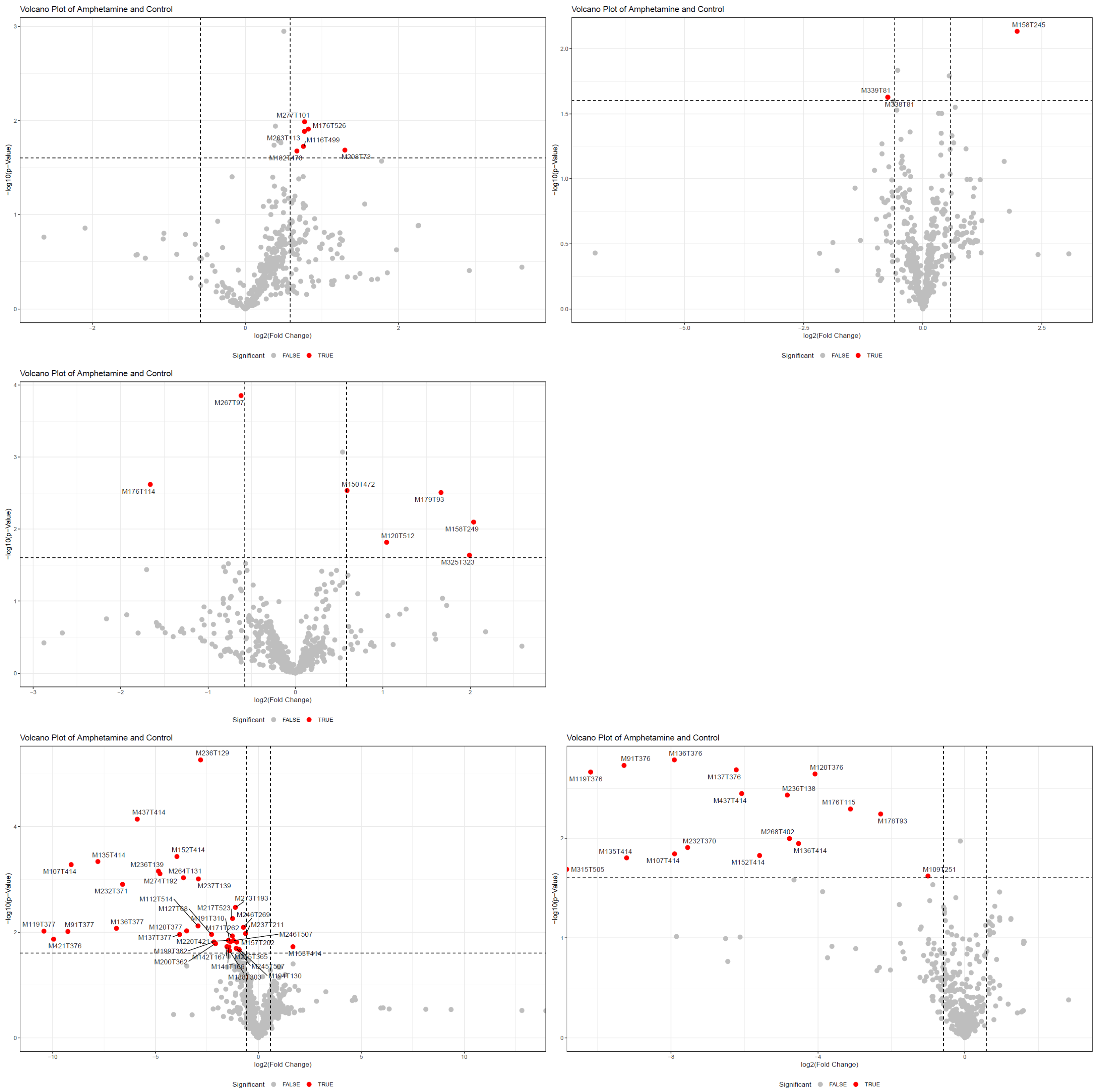

Fig. S1. Results of volcano plot for plasma and urine samples after analysis using normal phase chromatography and positive ionization mode. **a** = plasma 1 h; **b** = plasma 2 h; **c** = plasma 8 h; **d** = urine 8 h; **e** = urine 24 h.

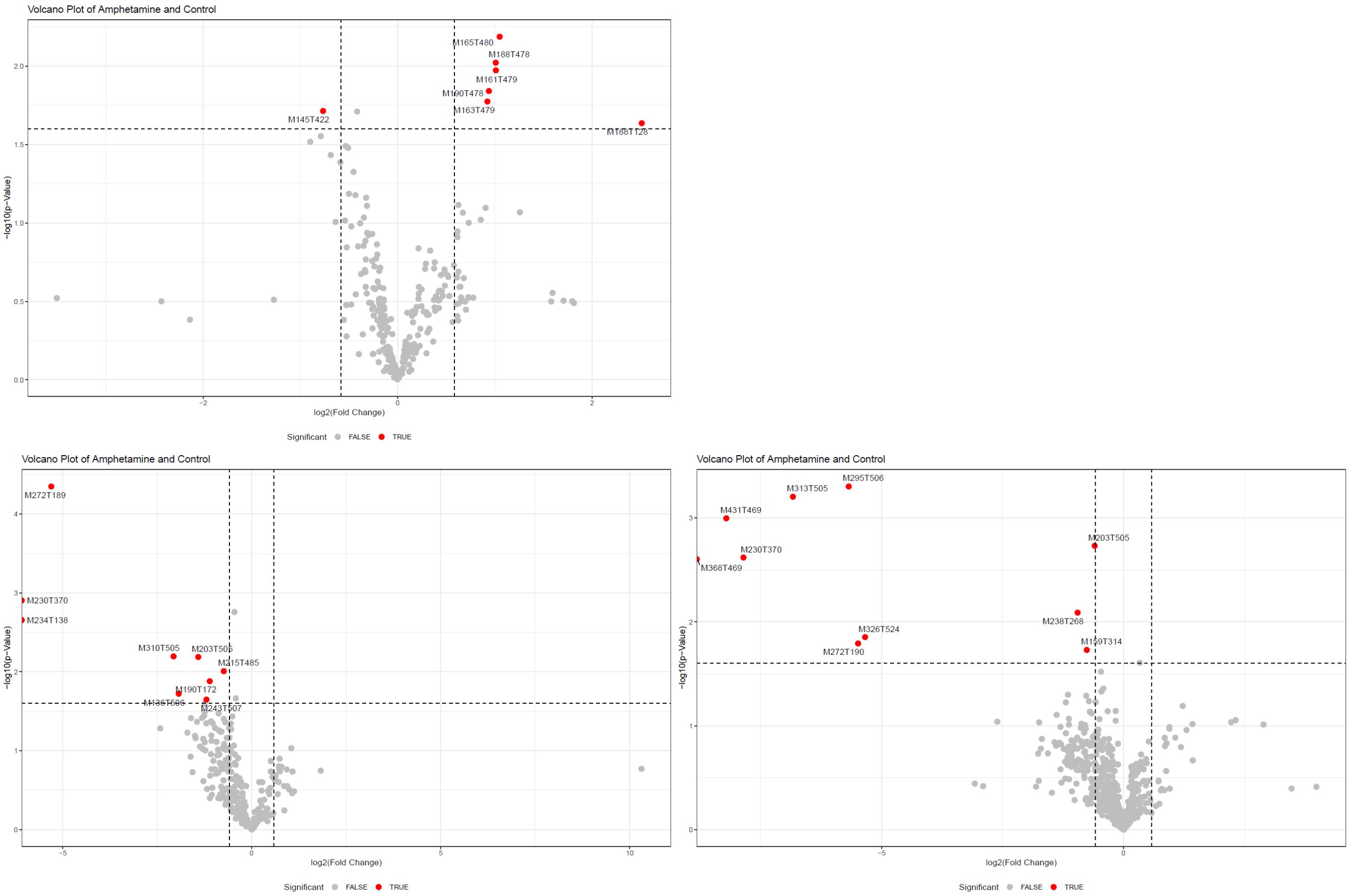

Fig. S2. Results of volcano plot for plasma and urine samples after analysis using normal phase chromatography and negative ionization mode. **a** = plasma 2 h; **b** = urine 8 h; **c** = urine 24 h.

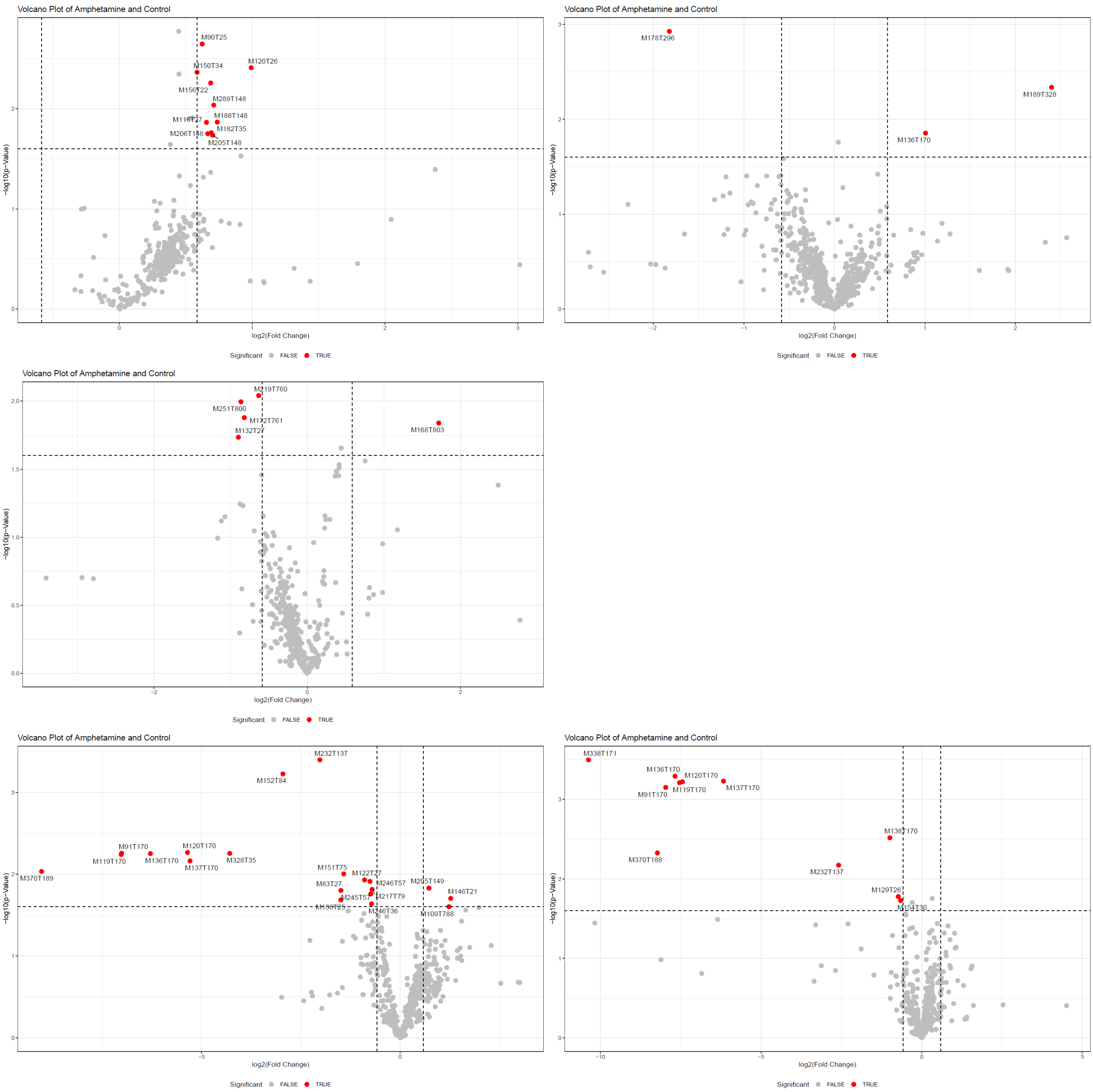

Fig. S3. Results of volcano plot for plasma and urine samples after analysis using reversed phase chromatography and positive ionization mode. **a** = plasma 1 h; **b** = plasma 2 h; **c** = plasma 8 h; **d** = urine 8 h; **e** = urine 24 h.

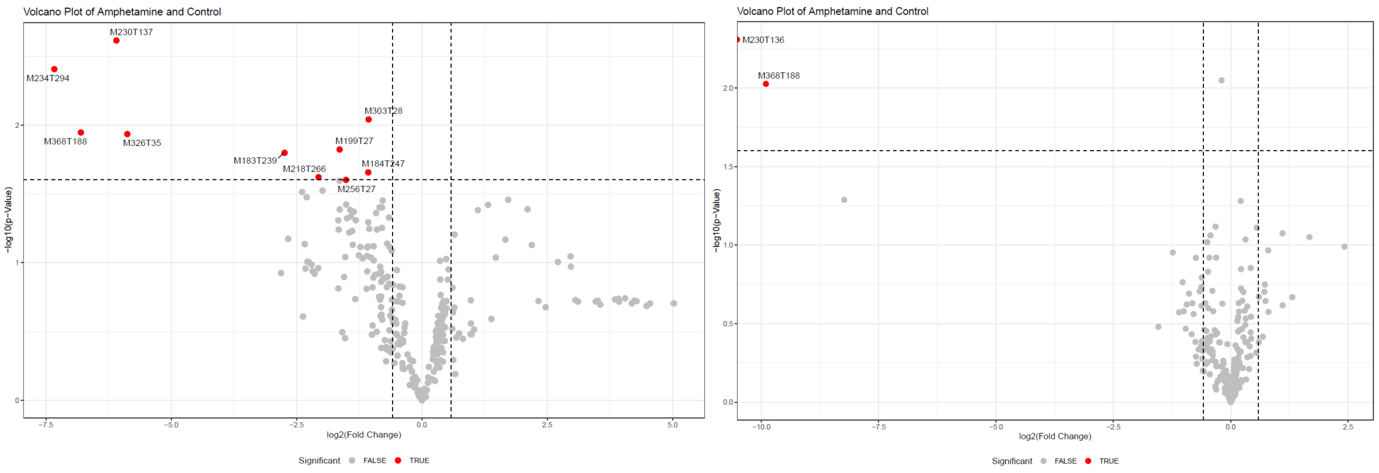

Fig. S4. Results of volcano plot for urine samples after analysis using reversed phase chromatography and negative ionization mode. **a** = urine 8 h; **b** = urine 24 h.

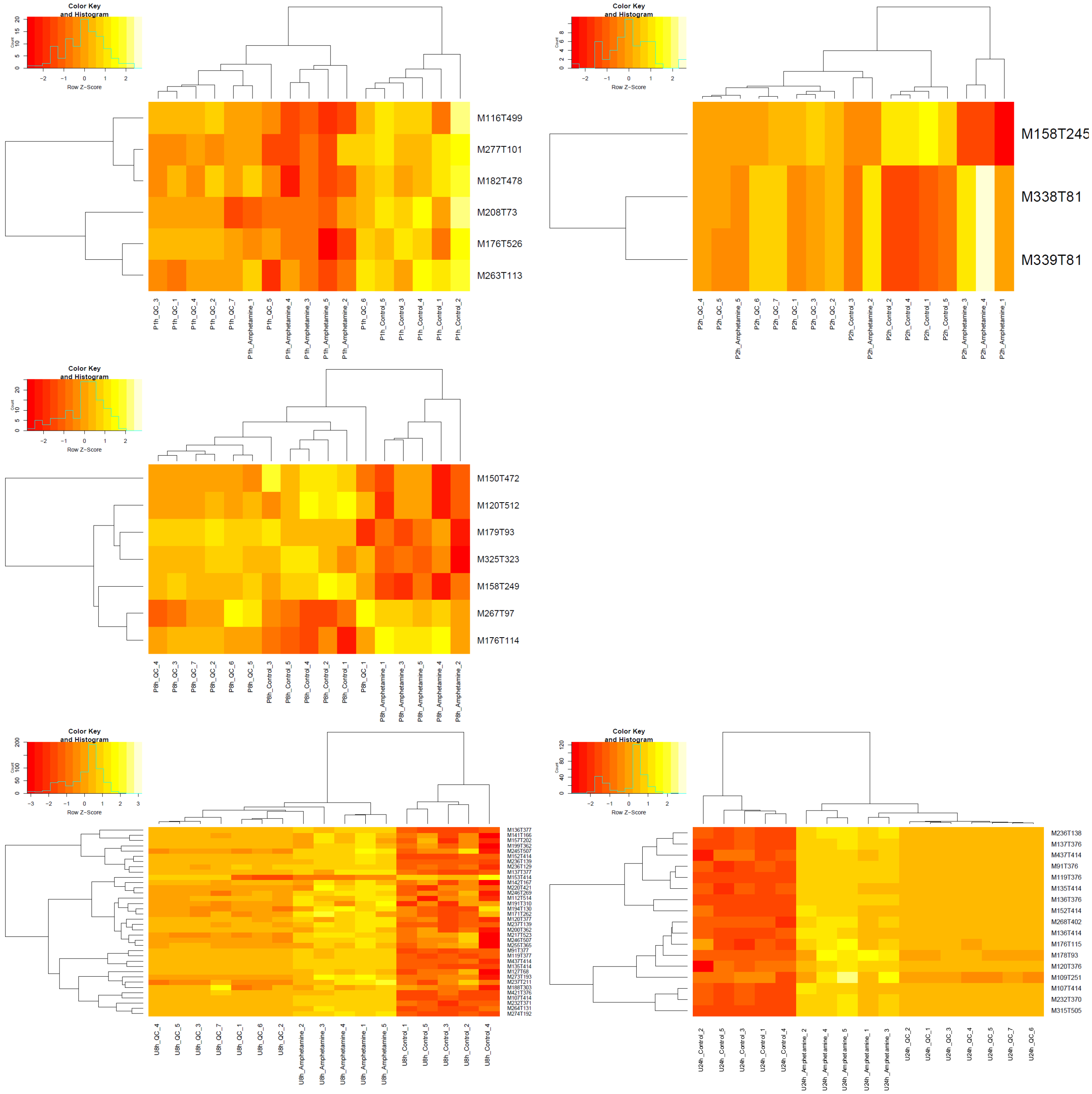

Fig. S5. Results of heat map of hierarchical clustering for plasma and urine samples after analysis using normal phase chromatography and positive ionization mode. **a** = plasma 1 h; **b** = plasma 2 h; **c** = plasma 8 h; **d** = urine 8 h; **e** = urine 24 h.

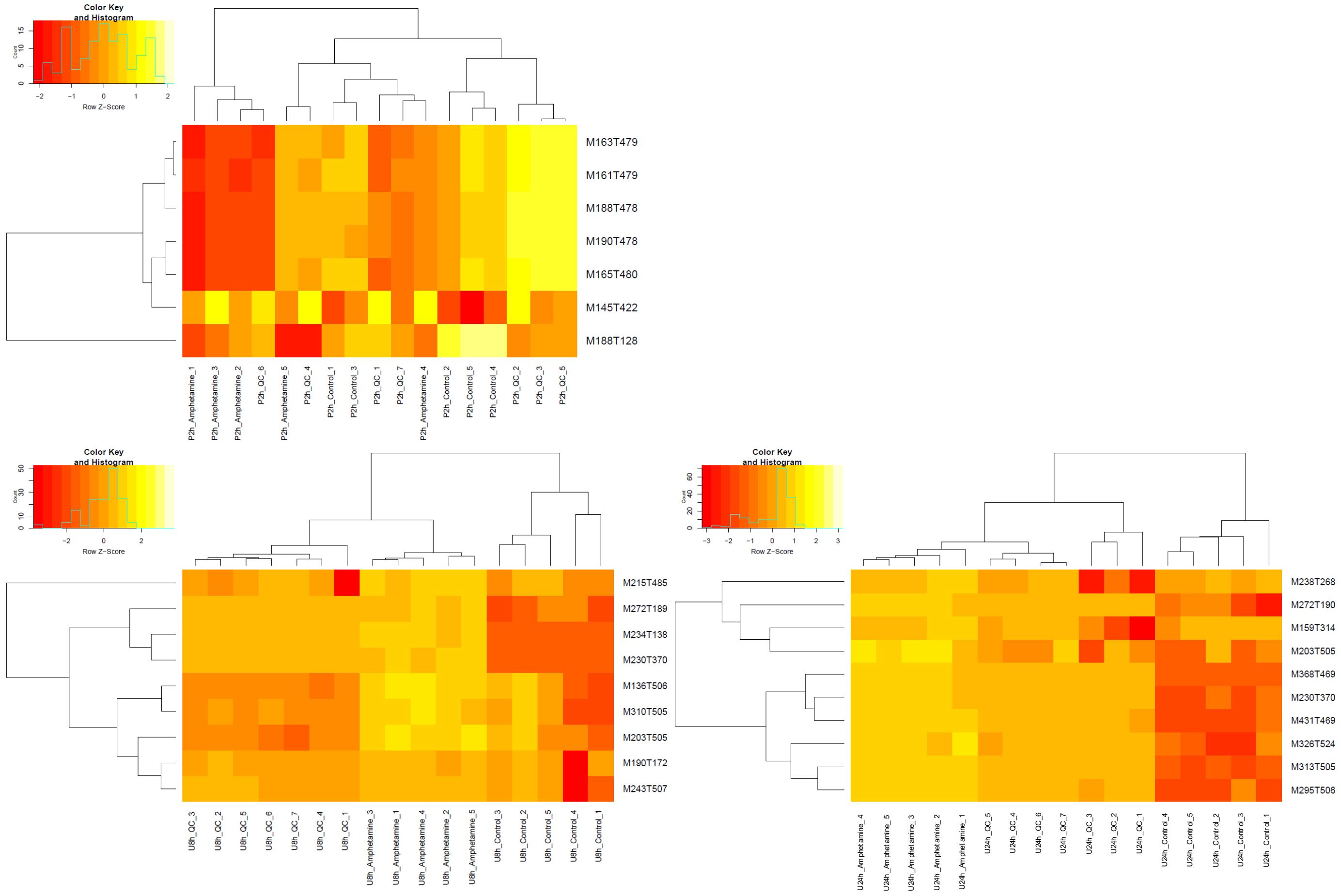

Fig. S6. Results of heat map of hierarchical clustering for plasma and urine samples after analysis using normal phase chromatography and negative ionization mode. **a** = plasma 2 h; **b** = urine 8 h; **c** = urine 24 h.

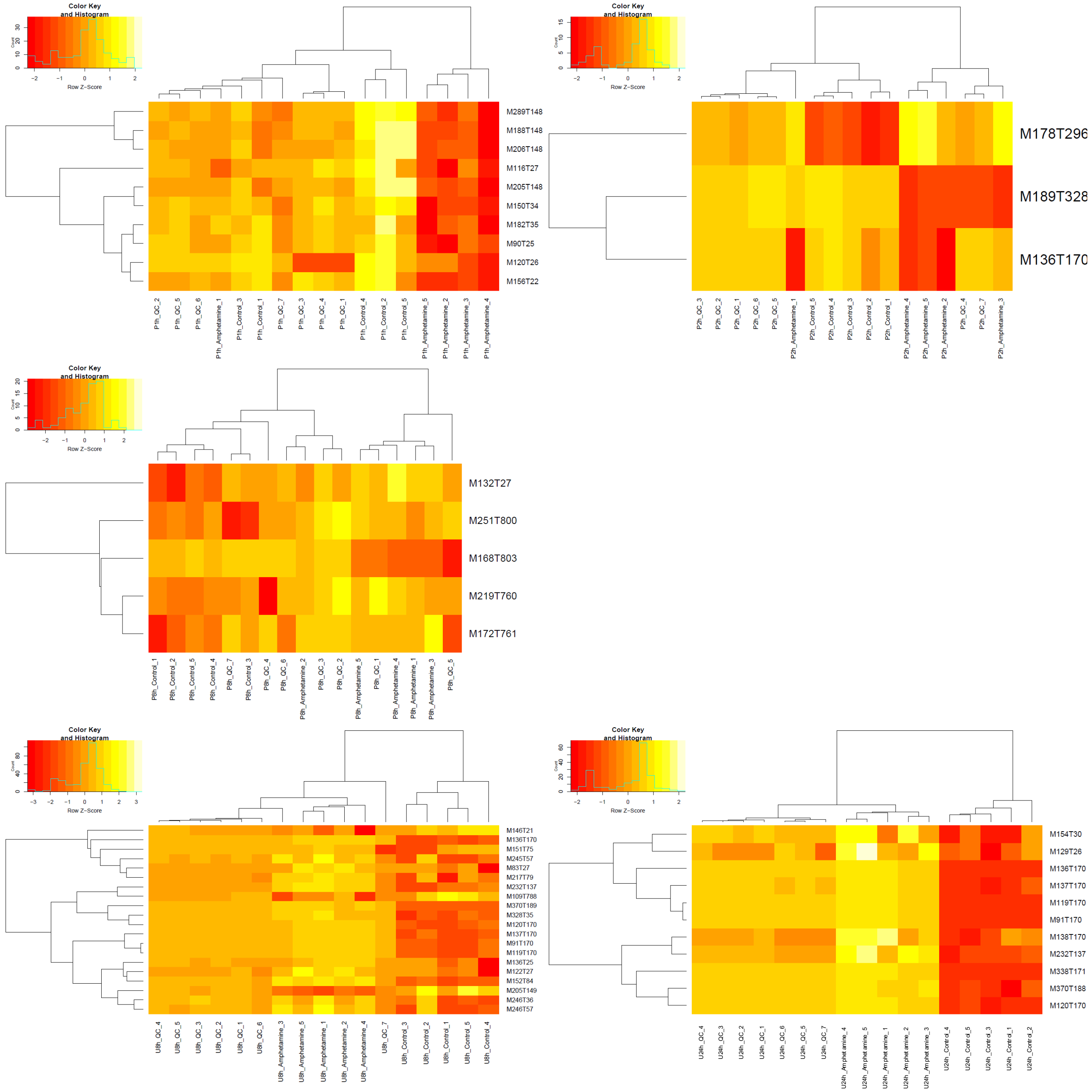

Fig. S7. Results of heat map of hierarchical clustering for plasma and urine samples after analysis using reversed phase chromatography and positive ionization mode. **a** = plasma 1 h; **b** = plasma 2 h; c = plasma 8 h; **d** = urine 8 h; **e** = urine 24 h.

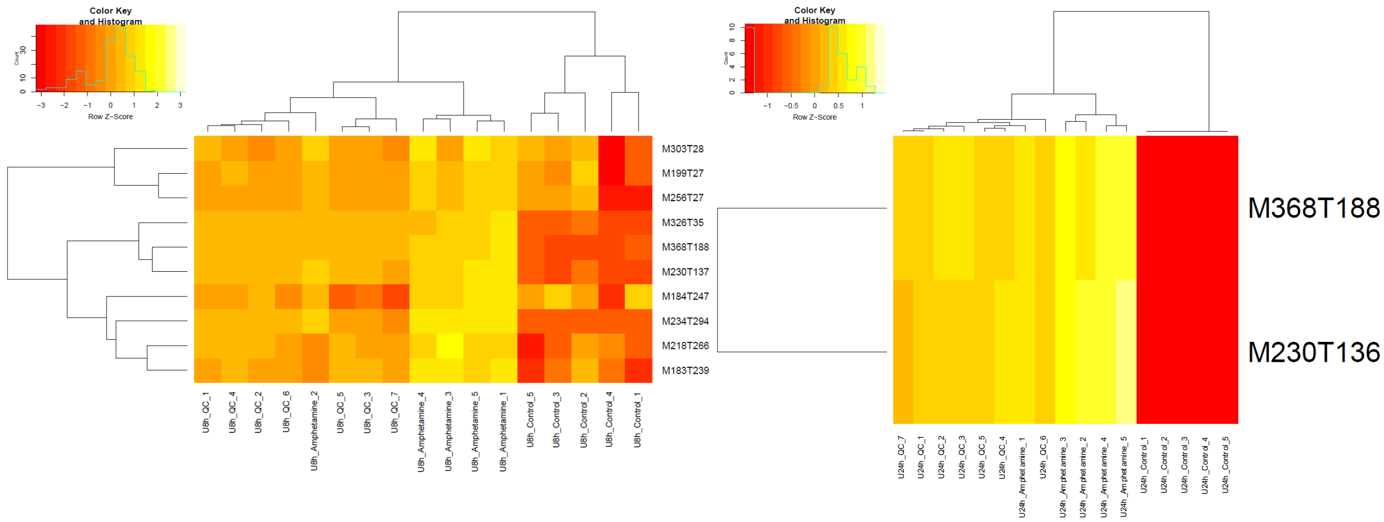

Fig. S8. Results of heat map of hierarchical clustering for urine samples after analysis using reversed phase chromatography and negative ionization mode. **a** = urine 8 h; **b** = urine 24 h.

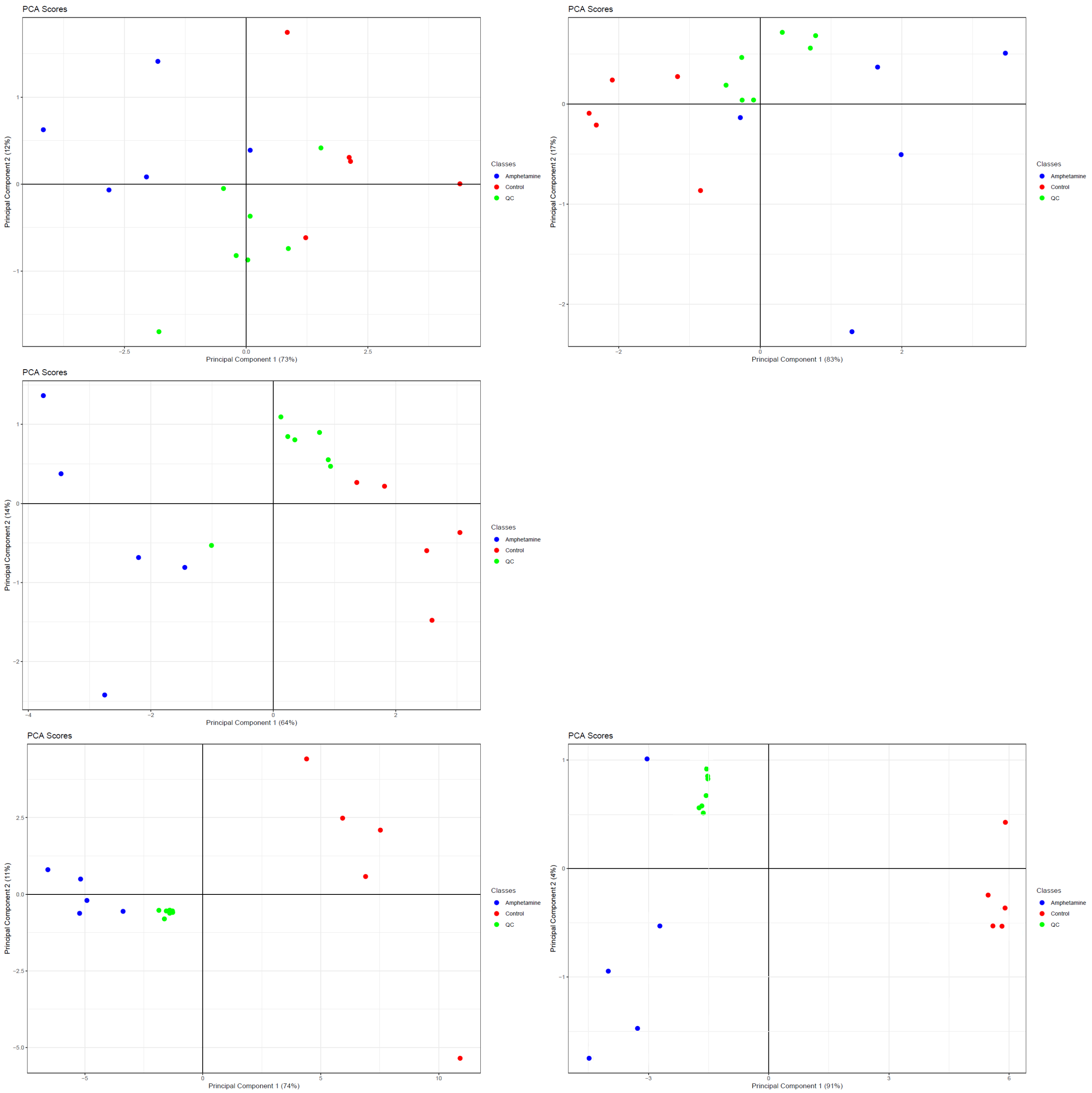

Fig. S9. Results of scores of principal component analysis for plasma and urine samples after analysis using normal phase chromatography and positive ionization mode. **a** = plasma 1 h; **b** = plasma 2 h; **c** = plasma 8 h; **d** = urine 8 h; **e** = urine 24 h.

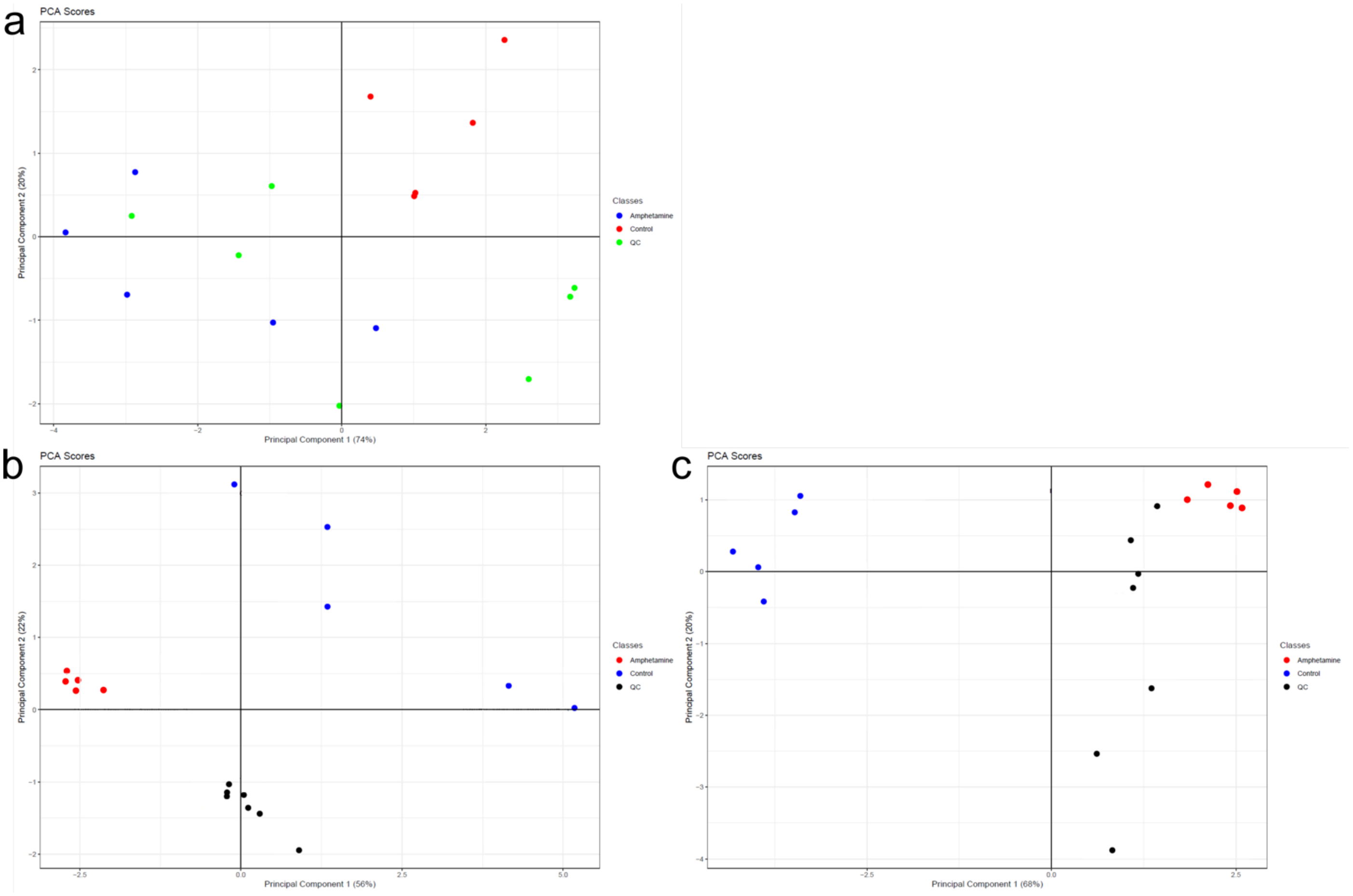

Fig. S10. Results of scores of principal component analysis for plasma and urine samples after analysis using normal phase chromatography and negative ionization mode. **a** = plasma 2 h; **b** = urine 8 h; **c** = urine 24 h.

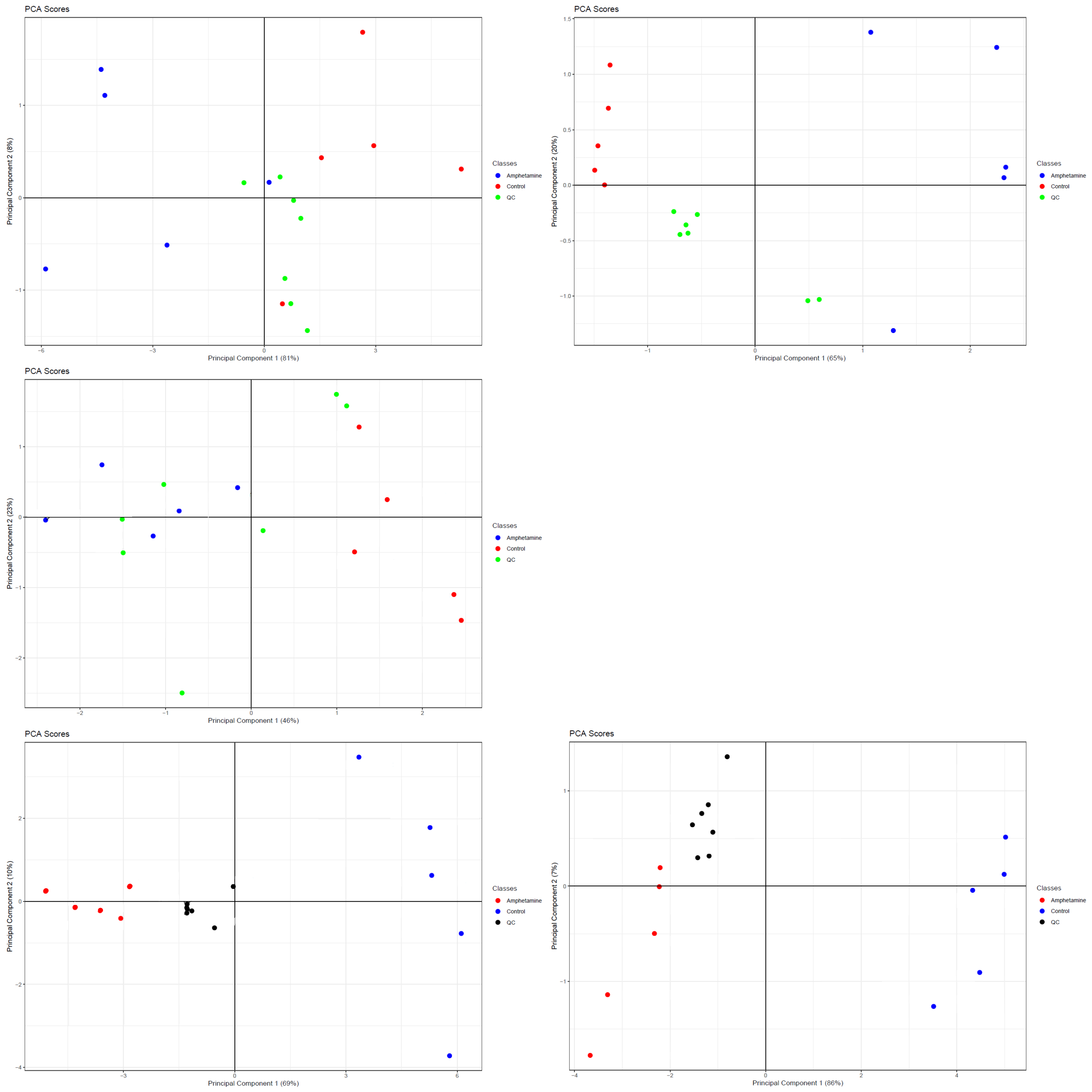

Fig. S11. Results of scores of principal component analysis for plasma and urine samples after analysis using reversed phase chromatography and positive ionization mode. **a** = plasma 1 h; **b** = plasma 2 h; **c** = plasma 8 h; **d** = urine 8 h; **e** = urine 24 h.

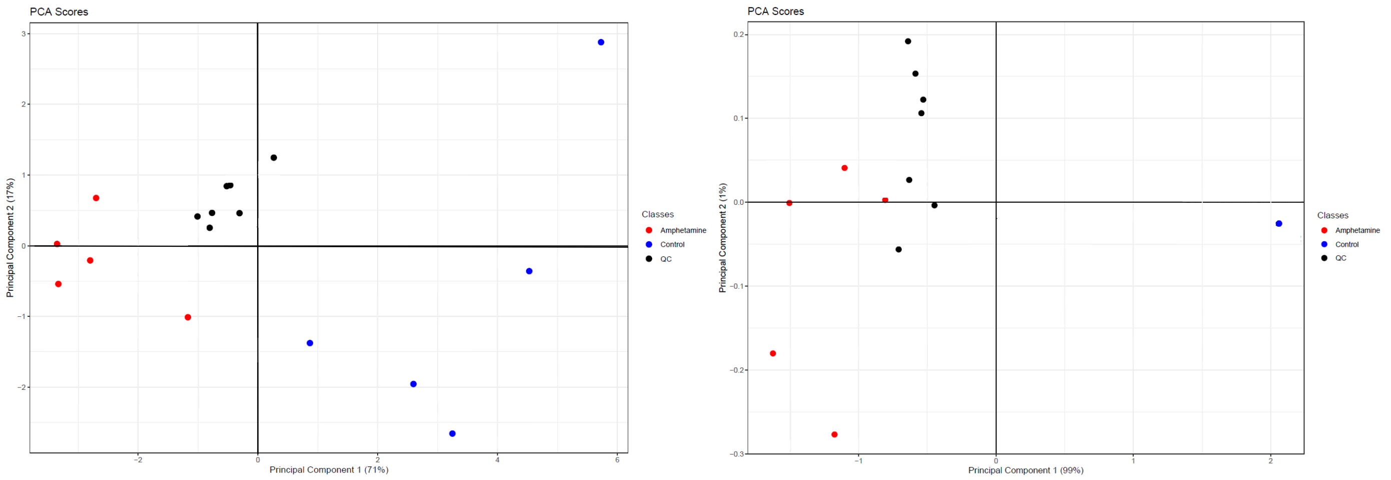

Fig. S12. Results of scores of principal component analysis for urine samples after analysis using reversed phase chromatography and negative ionization mode. **a** = urine 8 h; **b** = urine 24 h.

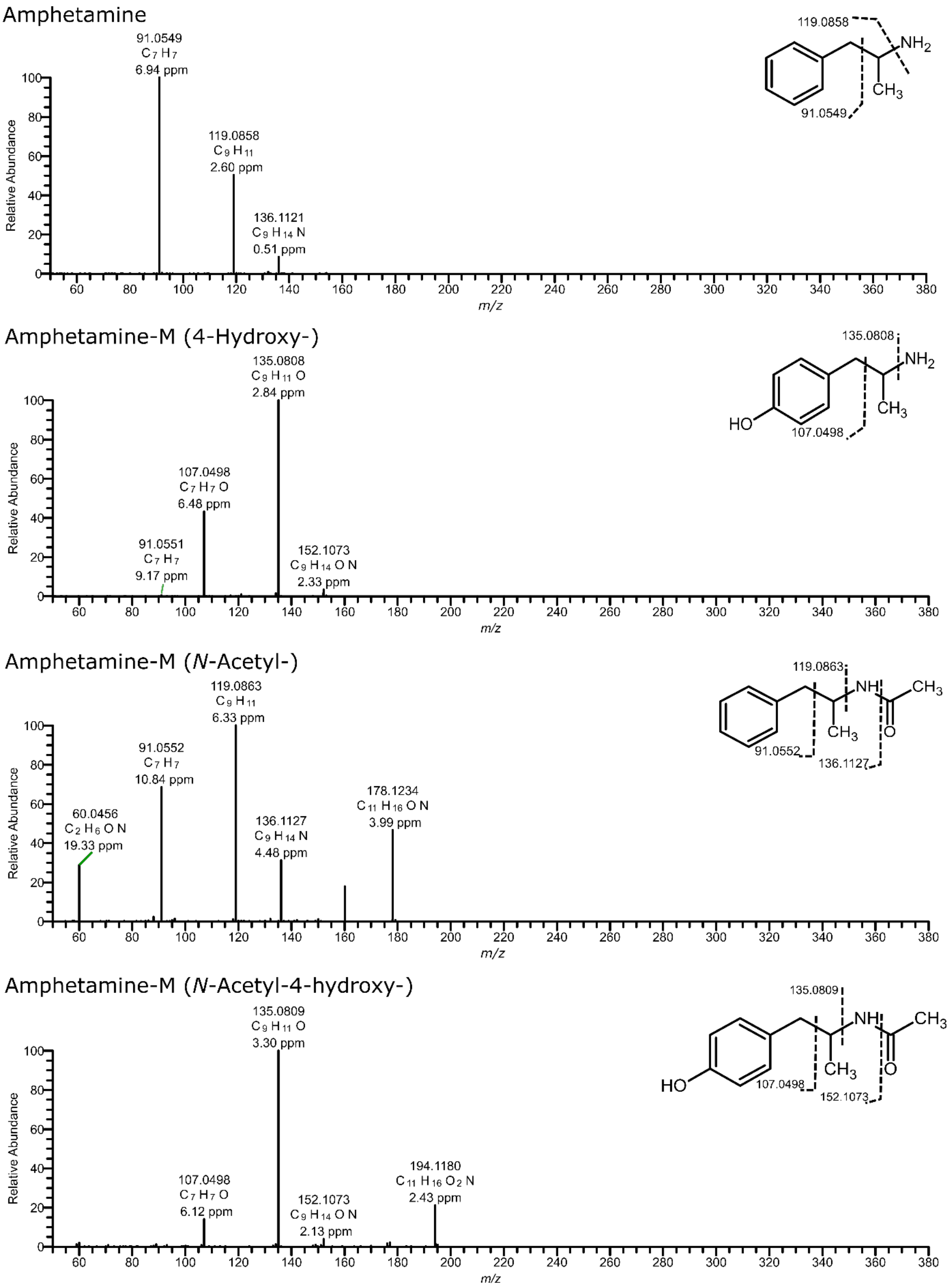

Fig. S13. LC-HRMS/MS spectra of amphetamine metabolites. Fragments with accurate mass, calculated elemental formula, and mass error value in parts per million (ppm).

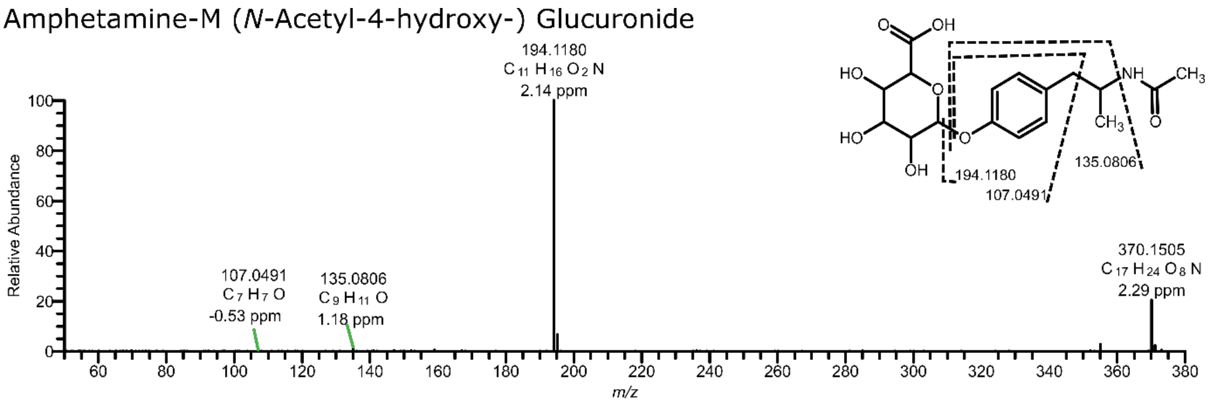

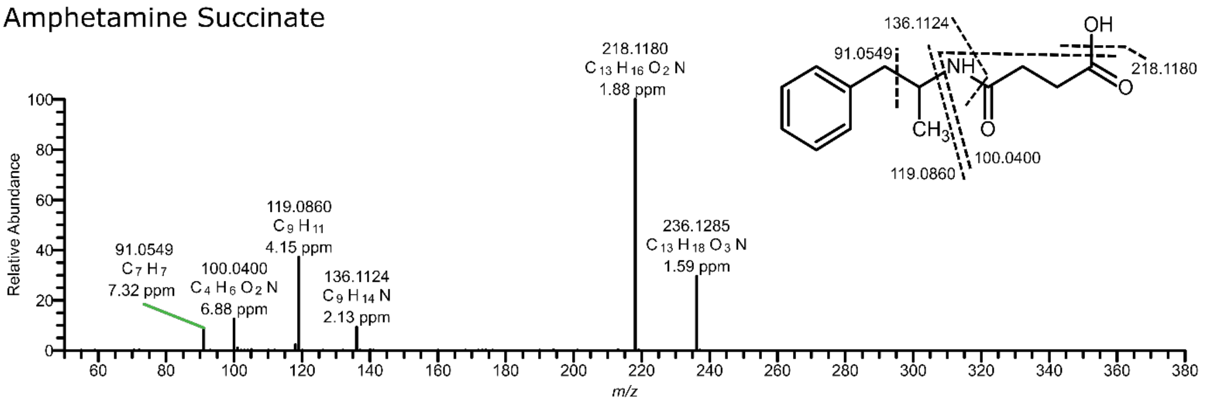

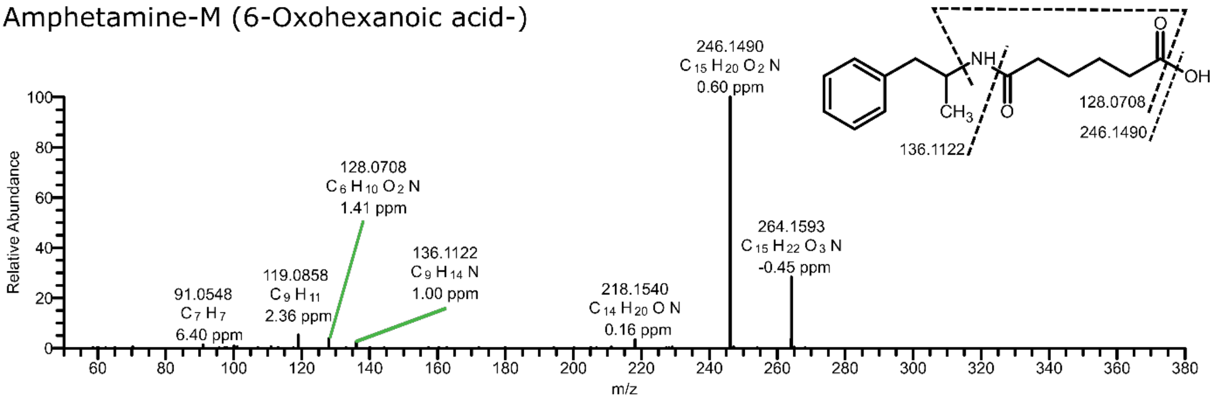

Fig. S13. Continued.
